# Autologous Blood Patch Pleurodesis for Persistent Pneumothorax in Emphysematous Lung: Salvage Success After Chemical Failure

**DOI:** 10.7759/cureus.87349

**Published:** 2025-07-05

**Authors:** Ernesto P Quinones-Gonzalez, Belissa A Lopez-Pena, Greisha Gonzalez-Santiago, Ricardo A Hernandez, Luis O Gerena-Montano

**Affiliations:** 1 Internal Medicine, San Juan City Hospital, San Juan, USA; 2 Pulmonary and Critical Care Medicine, San Juan City Hospital, San Juan, USA

**Keywords:** autologous blood patch pleurodesis, cost-effective intervention, emphysema, persistent air leak, pneumothorax, talc pleurodesis alternative

## Abstract

Persistent air leaks (PALs) present a significant challenge in patients with underlying structural lung abnormalities, such as emphysema. Conventional interventions, including chemical pleurodesis, may be ineffective or poorly tolerated in this population. We present a case of a high-risk patient with refractory pneumothorax successfully managed with autologous blood patch pleurodesis (ABPP). This approach demonstrated rapid resolution of air leak and complete lung re-expansion without surgical intervention. The case underscores ABPP's potential as a safe, bedside alternative in selected patients, particularly those with incomplete pleural apposition or high operative risk.

## Introduction

Persistent air leaks occur in up to 30% of patients with secondary spontaneous pneumothorax, particularly in those with underlying emphysema or bullous disease [1,2]. Talc pleurodesis, while widely used, is associated with complications such as pleuritic pain, fever, and, in rare cases, acute respiratory distress syndrome (ARDS) [3]. Furthermore, its efficacy is often reduced in patients with incomplete lung expansion [4]. Pleurodesis is the process of inducing fibrosis and adherence between the visceral and parietal pleura to eliminate the pleural space and prevent recurrent pneumothorax. Autologous blood patch pleurodesis (ABPP), first described by C.L. Robinson in 1987, is increasingly recognized as a safe, minimally invasive, and cost-effective alternative [2,5]. It promotes pleural adhesion through a combination of mechanical sealing and inflammation induced by autologous blood. This case aims to illustrate the efficacy of ABPP in a patient with high surgical risk and incomplete pleural apposition.

## Case presentation

**Figure 1:**
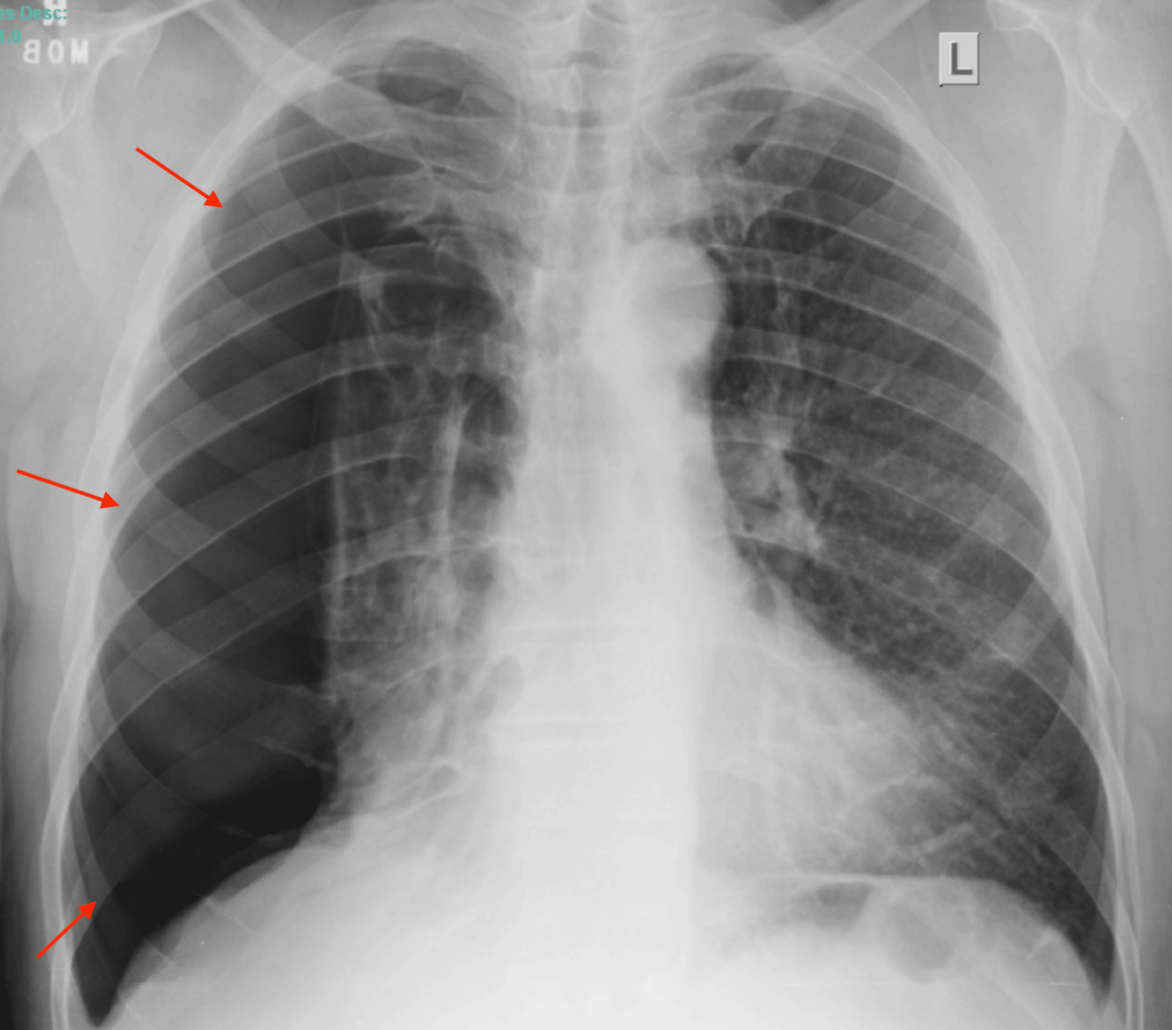
Pre-intervention chest radiograph showing large right-sided pneumothorax.

We present a 64-year-old man with a known history of severe chronic obstructive pulmonary disease and paraseptal bullous emphysema, not on home oxygen therapy, who presented to the emergency department with acute-onset right-sided pleuritic chest pain and progressive dyspnea while at rest. He denied fever, cough, or trauma. On examination, he was tachypneic with a respiratory rate of 28 breaths per minute, oxygen saturation of 88% on room air, blood pressure of 132/78 mmHg, and heart rate of 104 bpm. He exhibited decreased breath sounds on the right. A chest radiograph (Figure 1) revealed a large right-sided pneumothorax occupying approximately 70% of the hemithorax. A 14-Fr pleural catheter was inserted, resulting in partial lung re-expansion and symptomatic improvement.

**Figure 2:**
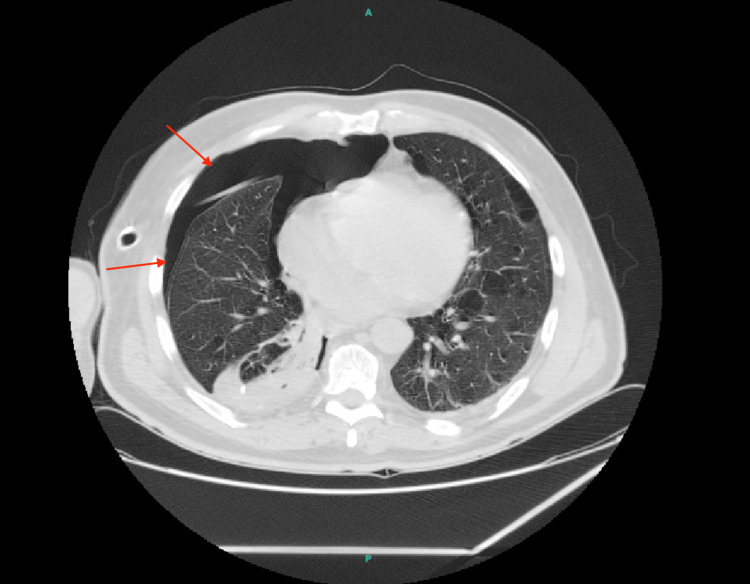
CT showing emphysematous bullae and pneumothorax with poor pleural apposition.

On day three of hospitalization, the catheter was inadvertently dislodged, and a standard chest tube was reinserted, yet a persistent air leak (PAL) was observed. The air leak was continuous, as evidenced by ongoing bubbling in the water seal chamber during both inspiration and expiration. Repeat chest imaging confirmed residual pneumothorax and worsening subcutaneous emphysema. On day five, chemical pleurodesis was attempted using iodine-based Lupol instilled via the chest tube, but the air leak persisted. A contrast-enhanced CT of the chest (Figure 2) demonstrated extensive paraseptal emphysema, multiple bullae, and poor pleural apposition, raising concern for refractory air leak in the context of structural lung disease.

Given the patient’s high surgical risk, due to limited pulmonary reserve, severe paraseptal emphysema with poor pleural apposition, and overall frailty, the multidisciplinary team elected to proceed with autologous blood patch pleurodesis on day seven. Sixty milliliters of the patient’s peripheral venous blood were drawn and mixed with 20 mL of sterile saline, then instilled into the pleural space via the chest tube. The patient was rotated every 30 minutes for over two hours to facilitate even distribution.

**Figure 3:**
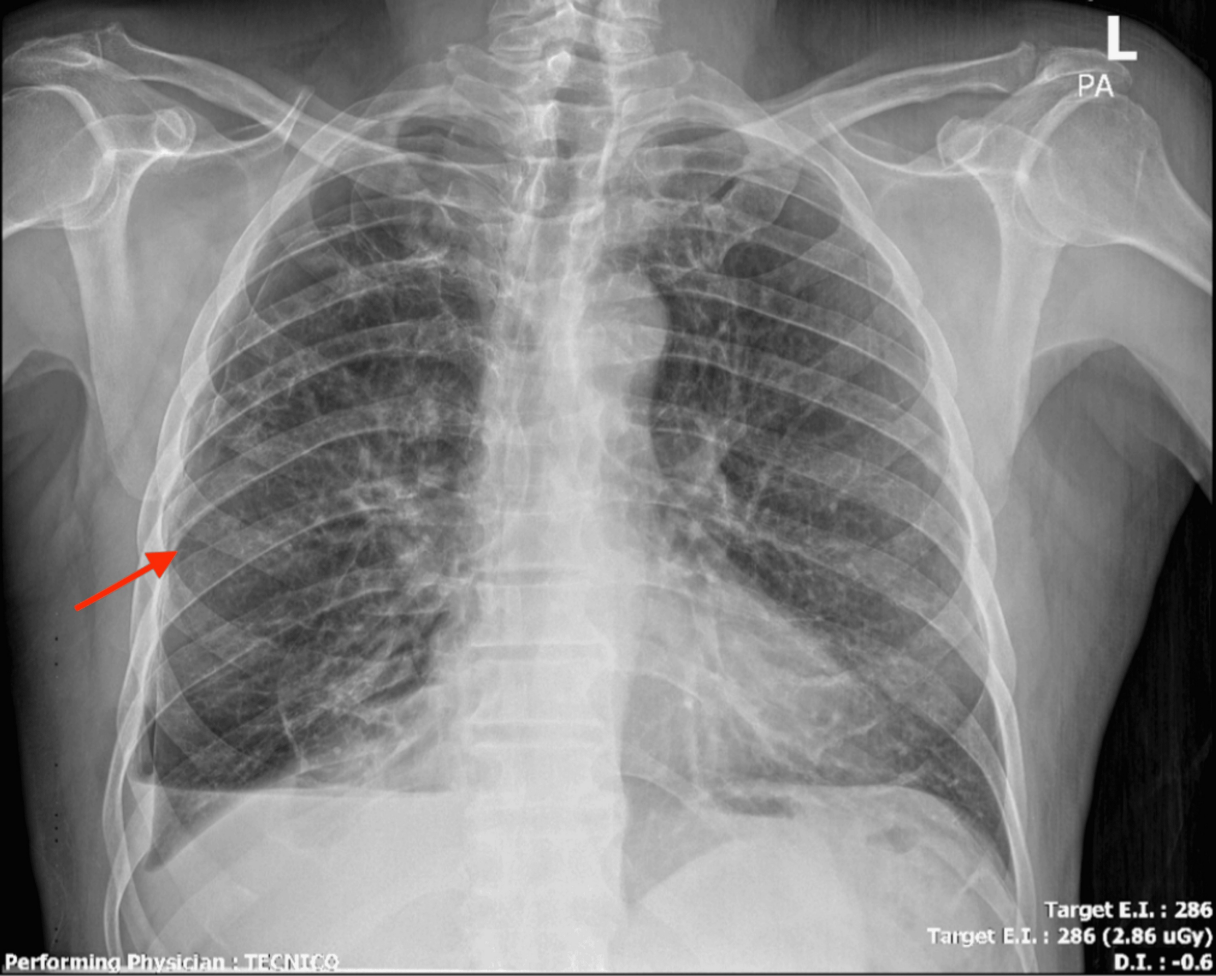
Post-ABPP chest radiograph showing lung re-expansion and resolution of pneumothorax. ABPP: Autologous blood patch pleurodesis

**Figure 4:**
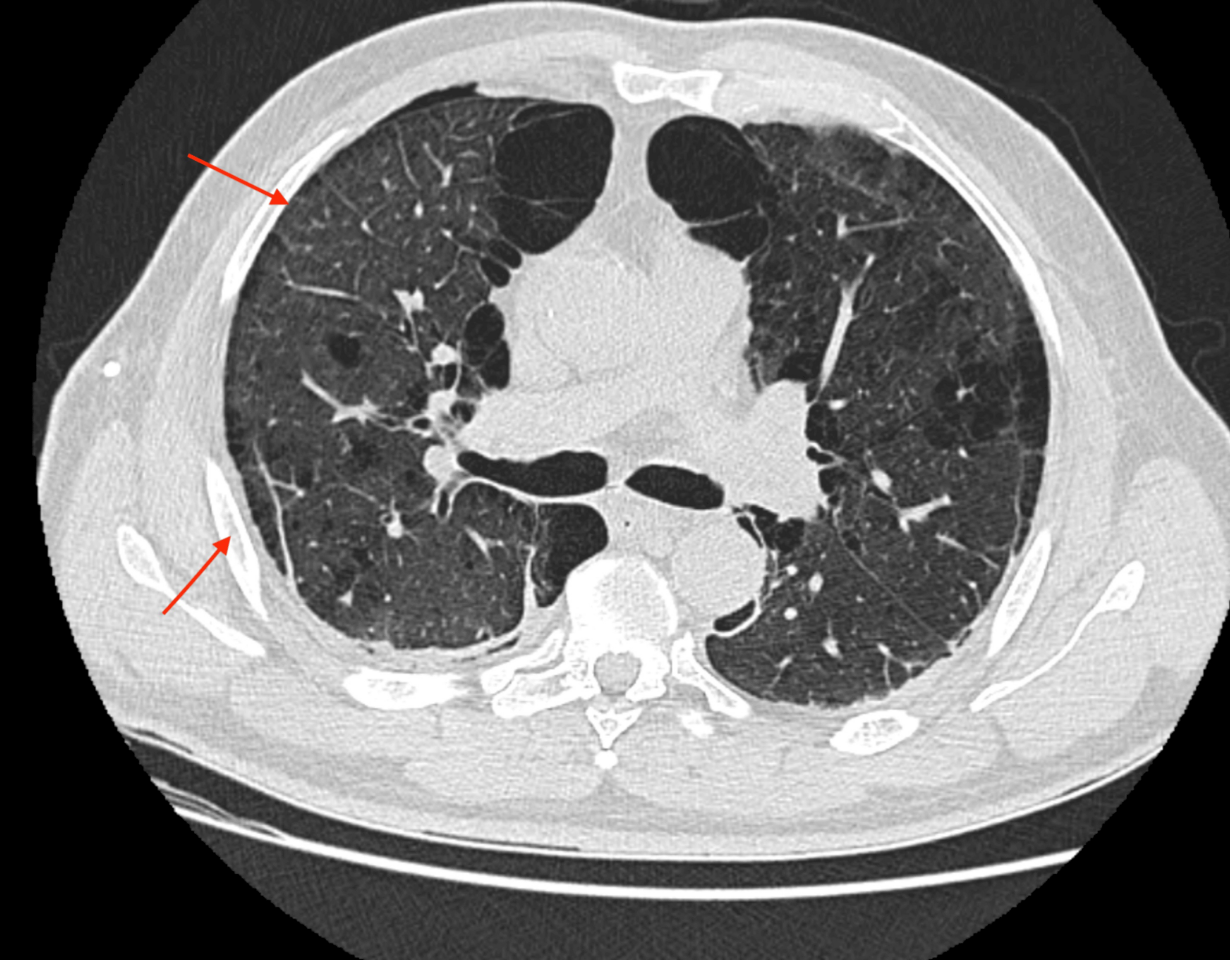
Follow-up CT confirming absence of pneumothorax and stable emphysema. CT: Computed tomography

The air leak ceased within 48 hours. The chest tube was removed on day nine, and the patient was discharged home on day 10 in stable condition. At the two-week outpatient follow-up, he remained asymptomatic with no evidence of recurrence. A follow-up chest radiograph (Figure 3) and computed tomography (Figure 4) obtained post-discharge confirmed full re-expansion of the lung and resolution of pneumothorax, with only a minimal residual anterior pneumothorax observed on initial post-procedure imaging, which resolved completely on subsequent scans.

## Discussion

Persistent pneumothorax in patients with structural lung disease, such as emphysema, presents a clinical and therapeutic challenge. Traditional interventions, including talc pleurodesis, have long been regarded as the standard of care due to their efficacy in inducing pleural adhesion. However, their use is not without risk, particularly in vulnerable patients with comorbidities. Talc pleurodesis has been associated with adverse events such as fever, chest pain, empyema, and, in rare cases, ARDS, especially in those with underlying interstitial lung abnormalities or older age [1].

ABPP induces pleurodesis through both mechanical and inflammatory mechanisms. The instilled autologous blood creates an immediate physical barrier that seals the site of the air leak. Concurrently, cytokines and growth factors released from the blood stimulate local inflammation, driving fibroblast migration and collagen deposition. This process leads to permanent pleural adhesion, mimicking the outcome of chemical pleurodesis but with lower associated morbidity. In contrast, talc pleurodesis relies solely on chemical pleuritis and subsequent fibrosis. It is most effective when pleural surfaces are fully opposed, an outcome often unachievable in patients with incomplete lung expansion due to underlying emphysematous changes or trapped lung physiology [2].

Our patient, who demonstrated poor pleural apposition on imaging, achieved complete resolution following ABPP. This highlights one of the key advantages of ABPP: efficacy even when full pleural contact is not attainable. In this case, cessation of the air leak was observed within 48 hours of the procedure, with subsequent re-expansion of the lung and early hospital discharge. Rapid control of air leaks can shorten hospital stays, reduce infection risk, and improve overall patient comfort [3-5].

Autologous blood patch pleurodesis (ABPP) offers a viable, bedside alternative to surgical interventions such as video-assisted thoracoscopic surgery (VATS) or thoracotomy, which carry increased risk in patients with limited pulmonary reserve or significant comorbidities. A direct comparison of pleurodesis techniques, including autologous blood, talc, and chemical agents, is provided in Table 1, underscoring ABPP’s favorable safety profile, low cost, and effectiveness even in cases of incomplete lung expansion. In addition to avoiding surgical morbidity, ABPP may improve quality of life by shortening chest tube duration, reducing dyspnea, and minimizing hospital-related stress, key considerations for patients with advanced chronic obstructive pulmonary disease (COPD) or emphysema [6].

**Table 1:** Comparison of pleurodesis methods

Aspect	Autologous Blood Patch (ABPP)	Talc Pleurodesis	Chemical Pleurodesis (e.g., iodine, doxycycline)	Comments
Mechanism	Clot sealing + autologous inflammation	Chemical pleuritis and fibrosis	Chemical pleuritis and fibrosis	ABPP works via mechanical and inflammatory pathways
Complication Rate	Low (pain <1%, fever ~12%)	Higher (pain ~30–35%, fever ~38%)	Moderate (pain ~15–25%, fever ~20–30%)	ABPP, typically, better tolerated in high-risk patients
Success Rate	72–94%	69–92%	60–85%	Comparable, but varies by study and lung expansion
Cost	Minimal (autologous blood)	Moderate (sterile talc)	Low to moderate (drug dependent)	ABPP most cost-effective at bedside
Effectiveness in Incomplete Expansion	Yes	Often ineffective	Often ineffective	ABPP can succeed without full pleural apposition
Setting	Bedside	OR or procedural suite	Bedside or procedural suite	ABPP feasible in ICU/ward without OR access

Given its low complication rate, procedural simplicity, and favorable outcomes, ABPP deserves consideration not only as salvage therapy but as a potential first-line modality in selecting high-risk patients. The trial by Metintas et al. demonstrated significantly lower rates of pain (0% vs. 34.5%) and fever (12% vs. 37.9%) in patients undergoing ABPP compared to talc pleurodesis [2]. This improved tolerability is especially important in patients with frailty, advanced age, or comorbid conditions. Although ABPP is generally safe, rare complications have been reported. These include tension pneumothorax or tamponade when large blood volumes are used or small-caliber catheters become obstructed [7,8]. To mitigate such risks, the use of large-bore chest tubes and the immediate availability of resuscitation equipment are recommended. While infection is rare, there is a theoretical risk of empyema or contamination. Strict aseptic techniques must be followed when drawing and instilling autologous blood, and appropriate patient monitoring should be in place. Importantly, the mechanism of ABPP is less dependent on full pleural apposition, making it particularly suitable for patients with trapped lung physiology or incomplete lung expansion, scenarios in which talc and chemical pleurodesis are often ineffective. As such, ABPP should not be viewed solely as a salvage option but rather as a potential first-line intervention in carefully selected patients where traditional modalities are contraindicated or fail.

As previously discussed, ABPP demonstrated efficacy comparable to talc pleurodesis in the Metintas et al. noninferiority trial [5]. Similar success rates (~72-94%) have been reported across other small cohort studies, reinforcing its role in managing persistent air leaks [2,3,9]. ABPP is highly cost-effective. It requires no specialized agents, can be performed at the bedside, and reduces the need for operative intervention or prolonged hospitalization. This makes it particularly attractive in resource-limited settings or community hospitals without surgical thoracic services. Surgical options such as bullectomy and mechanical pleurodesis remain definitive treatments in fit patients. However, studies suggest that ABPP achieves comparable efficacy in sealing air leaks without the morbidity associated with anesthesia or thoracotomy, especially in emphysematous lungs where bullae are diffusely distributed [4,10].

Multiple case series and prospective evaluations have demonstrated that ABPP yields high success rates, often above 80%, in patients with persistent air leaks across various clinical scenarios, including postoperative, trauma-related, and spontaneous pneumothoraces. These studies consistently report low complication rates and rapid resolution times, even in patients with incomplete lung expansion or high surgical risk [9].

Success has been observed using a range of blood volumes, typically 50-250 mL, and favorable outcomes have been reported regardless of the etiology of the air leak. The reproducibility of results in both resource-rich and limited-resource settings underscores ABPP’s versatility and cost-effectiveness. Despite favorable outcomes, most available data are derived from small, retrospective, or nonrandomized studies. Variations in technique, volume of instilled blood, and patient selection criteria further complicate standardization. There is a clear need for multicenter randomized trials and formal inclusion of ABPP in pleural disease guidelines. Standardization of technique, patient selection, and timing will help define its optimal role in modern thoracic practice.

## Conclusions

Autologous blood patch pleurodesis (ABPP) is a safe, effective, and low-cost treatment option for managing persistent air leaks, particularly in patients with emphysematous lung disease and high surgical risk. In this case, ABPP led to the complete resolution of a refractory pneumothorax after the failure of chemical pleurodesis, enabling early discharge and avoiding surgical intervention. Compared to traditional agents such as talc, ABPP offers reduced complication rates, bedside feasibility, and effectiveness even in cases of incomplete lung re-expansion. These advantages make ABPP a valuable alternative in carefully selected patients and in resource-limited settings. We recommend greater awareness, clinical training, and formal inclusion of ABPP in pleural disease protocols. Further studies are needed to support its earlier use in high-risk patients with persistent air leaks.
